# Diverse DNA Sequence Motifs Activate Meiotic Recombination Hotspots Through a Common Chromatin Remodeling Pathway

**DOI:** 10.1534/genetics.119.302679

**Published:** 2019-09-11

**Authors:** Tresor O. Mukiza, Reine U. Protacio, Mari K. Davidson, Walter W. Steiner, Wayne P. Wahls

**Affiliations:** * Department of Biochemistry and Molecular Biology, University of Arkansas for Medical Sciences, Little Rock, Arkansas 72205-7199; † Department of Biology, Niagara University, Lewiston, New York 14109

**Keywords:** meiosis, genetic recombination, nucleosome, chromatin remodeling, epigenetics

## Abstract

In meiosis, multiple different DNA sequence motifs help to position homologous recombination at hotspots in the genome. How do the seemingly disparate *cis*-acting regulatory modules each promote locally the activity of the basal recombination machinery? We defined molecular mechanisms of action for five different hotspot-activating DNA motifs (*M26*, *CCAAT*, *Oligo-C*, *4095*, *4156*) located independently at the same site within the *ade6* locus of the fission yeast *Schizosaccharomyces pombe*. Each motif promoted meiotic recombination (*i.e.*, is active) within this context, and this activity required the respective binding proteins (transcription factors Atf1, Pcr1, Php2, Php3, Php5, Rst2). High-resolution analyses of chromatin structure by nucleosome scanning assays revealed that each motif triggers the displacement of nucleosomes surrounding the hotspot motif in meiosis. This chromatin remodeling required the respective sequence-specific binding proteins, was constitutive for two motifs, and was enhanced meiotically for three others. Hotspot activity of each motif strongly required the ATP-dependent chromatin remodeling enzyme Snf22 (Snf2/Swi2), with lesser dependence on Gcn5, Mst2, and Hrp3. These findings support a model in which most meiotic recombination hotspots are positioned by the binding of transcription factors to their respective DNA sites. The functional redundancy of multiple, sequence-specific protein–DNA complexes converges upon shared chromatin remodeling pathways that help provide the basal recombination machinery (Spo11/Rec12 complex) access to its DNA substrates within chromatin.

## Abstract (precis)

Homologous recombination is induced to high levels in meiosis and is clustered at hotspots that regulate its frequency and distribution in the genome. By studying five different classes of DNA sequence-dependent recombination hotspots in the fission yeast...

MEIOSIS couples one round of DNA replication, high-frequency recombination between homologous chromosomes (homologs), and two rounds of chromosome segregation to produce haploid meiotic products. In most eukaryotes, recombination is required for the faithful segregation of homologs at the first meiotic division and it generates genetic diversity upon which natural selection can act (Székvölgyi and Nicolas 2009; Handel and Schimenti 2010).

The broadly conserved catalytic subunit of the basal meiotic recombination machinery, Spo11 (Rec12 in fission yeast), introduces DNA double-strand breaks (DSBs) that initiate, and are required for, meiotic recombination throughout the genome (Keeney *et al.* 1997; Gerton *et al.* 2000; Sharif *et al.* 2002; Buhler *et al.* 2007; Cromie *et al.* 2007; Kan *et al.* 2011). While DSB-initiated recombination can occur anywhere along chromosomes, it is clustered preferentially at hotspots that regulate its frequency and positioning in the genome (Smukowski and Noor 2011; Dluzewska *et al.* 2018; Tock and Henderson 2018). As is the case for transcription, *cis*-acting regulatory elements (transcription factor binding sites) help to localize the activity of the basal recombination machinery at its preferred sites of action (Wahls and Davidson 2010, 2012).

Allele-specific (*i.e.*, *cis*-acting) regulation of meiotic recombination was first reported for the *ade6-M26* hotspot of fission yeast (Gutz 1971). A single base pair substitution in the *ade6* locus created serendipitously a 7-bp DNA sequence motif (the *M26* DNA site, Table 1) that is essential for hotspot activity (Schuchert *et al.* 1991). Binding of the heterodimeric transcription factor Atf1-Pcr1 (originally called Mts1-Mts2) (Wahls and Smith 1994) to the *M26* DNA site increases recombination ∼20-fold, relative to control alleles of *ade6* that lack the *M26* DNA site (Wahls and Smith 1994; Kon *et al.* 1997, 1998; Gao *et al.* 2008). The Atf1-Pcr1-*M26* protein–DNA complex triggers chromatin remodeling in meiosis (Yamada *et al.* 2004; Hirota *et al.* 2007) and stimulates the catalysis of recombination-initiating DSBs by Rec12/Spo11 (Steiner *et al.* 2002). The hotspot-specific chromatin remodeling precedes the formation of DSBs and does not require Rec12 (Hirota *et al.* 2007), suggesting that chromatin remodeling functions upstream of (*i.e.*, likely promotes) the formation of DSBs at this hotspot.

**Table 1:** Hotspot-activating DNA sequence motifs and their binding proteins

Motif name	Motif sequence*^a^*	Binding proteins
*M26*	5′-ATGACGT-3′	Atf1-Pcr1
*CCAAT*	5′-CCAATCA-3′	Php2-Php3-Php5
*Oligo-C*	5′-CCCCGCAC -3′	Rst2
*4095*	5′-GGTCTRGACC-3′	Unknown
*4156*	5′-TCGGCCGA-3′	Unknown

a Experimentally defined core sequences that are sufficient to promote recombination locally; R = A or G. The base pair substitutions used to generate these DNA binding sites in the *ade6* gene and the DNA sequences of all other *ade6* alleles used in this study are provided in Table S2.

A subset of naturally occurring and artificially created *M26* DNA sites located elsewhere in the genome, within both coding and noncoding regions, promote recombination locally (Virgin *et al.* 1995; Steiner and Smith 2005). Interestingly, around three-quarters of the *M26* sites in the genome are not recombinogenic (Wahls and Davidson 2010), even though most of them are occupied by the Atf1-Pcr1 heterodimer (Kon *et al.* 1998; Eshaghi *et al.* 2010; Woolcock *et al.* 2012). Additional factors, such as nearby, *cis*-linked, DNA sequence elements (Zahn-Zabal *et al.* 1995), and, potentially, structural features such as chromatin loop-axis domains (Miyoshi *et al.* 2012; Yamada *et al.* 2018), are required for Atf1-Pcr1-*M26* complexes to promote recombination locally. Such “context-variable penetrance” (Wahls and Davidson 2012) also occurs for other known and inferred *cis*-acting regulatory factors of fission yeast and other species, including other recombinogenic DNA sequences (Mieczkowski *et al.* 2006; Myers *et al.* 2008; Steiner *et al.* 2011), histone PTMs (Brick *et al.* 2012; Yamada *et al.* 2013), open chromatin (Berchowitz *et al.* 2009; de Castro *et al.* 2011), and long noncoding RNAs (Wahls *et al.* 2008). Nevertheless, the subset of naturally occurring *M26* DNA sites that do promote recombination are implicated to help position ∼20% of all recombination in the fission yeast genome (Wahls and Davidson 2010).

A large, gain-of-function screen identified numerous short DNA sequences that promote meiotic recombination in fission yeast, and *M26* was among the consensus sequence motifs discovered, validating the approach (Steiner *et al.* 2009). Four additional, newly discovered consensus motifs (*CCAAT*, *Oligo-C*, *4095*, and *4156*) were subsequently refined functionally at single-nucleotide resolution by systematic base pair substitution analyses (Table 1) (Steiner *et al.* 2009, 2011; Foulis *et al.* 2018). As with *M26*-promoted recombination (Wahls and Smith 1994; Kon *et al.* 1997, 1998; Gao *et al.* 2008), the *CCAAT* and *Oligo-C* motifs are bound by transcription factors (Table 1) that are essential for hotspot activity (Steiner *et al.* 2009, 2011; Foulis *et al.* 2018). Notably, the proteins that bind to and activate these specific, sequence-dependent hotspots have no significant impact on the activation of heterologous sequence-dependent hotspots to which they do not bind (Steiner *et al.* 2009, 2011). Proteins that bind to DNA motifs *4095* and *4156* have not yet been identified. Nearly 200 additional hotspot-activating DNA sequences that were identified in the screen share no obvious homology with each other or with the defined motifs. Thus, ∼200 (and potentially more) short, distinct DNA sequence elements, and, by inference, their sequence-specific binding proteins, help to position recombination at hotspots in fission yeast—and, together, they have the potential to regulate all hotspots in the genome (Steiner *et al.* 2009, 2011; Wahls and Davidson 2010, 2012).

The DNA sequence-dependent regulation of meiotic recombination hotspots has also been reported for budding yeast, mice, and humans, and has been implicated by association in many other species. In budding yeast, deletions and insertions that contain transcription factor binding sites ablate and create hotspots, respectively, and the corresponding binding proteins are required for hotspot activity (White *et al.* 1991, 1993; Fan *et al.* 1995). The different protein–DNA complexes can function redundantly at the same locus, which can explain (Wahls and Davidson 2012) why ablating a transcription factor does not necessarily abolish hotspot activity near all of its binding sites (Mieczkowski *et al.* 2006; Zhu and Keeney 2015). Like the regulatory protein–DNA complexes of fission yeast and mammals, those of budding yeast display context-variable penetrance (Mieczkowski *et al.* 2006; Zhu and Keeney 2015). Nevertheless, genome-wide, ∼52% of DSB hotspots in budding yeast colocalize with DNA binding sites for 77 transcription factors (Pan *et al.* 2011). Extrapolation for the estimated 140–250 transcription factors (Hughes and de Boer 2013) suggests that transcription factor binding sites could account for the regulated positioning of most, if not all, hotspots in this organism.

Among the five well-characterized regulatory elements of fission yeast, *M26* DNA sites and Atf1-Pcr1 heterodimer contribute the most to recombination across the genome (Steiner *et al.* 2009, 2011; Wahls and Davidson 2010; Foulis *et al.* 2018). The homologous recombination activation (HRA) domain resides in Atf1 (Gao *et al.* 2008). The Atf1 ortholog Sko1 of budding yeast also seems to be quite recombinogenic, based on the very high frequency with which DSBs are directed to Sko1 binding sites (Pan *et al.* 2011). Other sequence-specific binding proteins proven to activate hotspots in fission yeast (Php2, Php3, Php5, Rst2) (Steiner *et al.* 2009, 2011) also have orthologs in budding yeast (Hap2, Hap3, Hap5, Adr1), and, in each case, DSBs are directed preferentially to their binding sites (Pan *et al.* 2011). Moreover, four of the regulatory DNA sequences discovered in fission yeast also promote recombination (*i.e.*, generate hotspots) when placed at a test locus in budding yeast (Steiner and Steiner 2012). Thus, the positioning of meiotic recombination hotspots by specific DNA sites and their binding proteins (transcription factors) is conserved between two species that are as evolutionarily distant from each other as either species is from human beings (Sipiczki 2000). Data from other taxa are also consistent with broad conservation of *cis*-acting regulatory mechanisms. For example, DNA sequence motifs implicated in helping position meiotic recombination in honeybees (Mougel *et al.* 2014) match, or are similar to, respectively, the *CCAAT* and *Oligo-C* motifs that are known to activate hotspots in fission yeast (Steiner *et al.* 2009, 2011).

How do seemingly disparate DNA sequences and their binding proteins each promote locally the activity of the basal recombination machinery? Here, we report that each of five distinct, well-defined, sequence-dependent recombination hotspots of fission yeast (Table 1) employs a common mechanism of function. They each trigger the remodeling of chromatin structure in meiosis, and, in each case, the ATP-dependent chromatin remodeling enzyme Snf22 (Snf2/Swi2) is a key effector of high frequency recombination.

## Materials and Methods

### Fission yeast husbandry

Genotypes of *S. pombe* strains used in this study are listed in Supplemental Material, Table S1 and the DNA sequences of all *ade6* alleles used are provided in Table S2. We use standard fission yeast genetic nomenclature (Kohli 1987) for wild-type genes (*e.g.*, *ade6*), variant alleles (*e.g.*, *ade6-M210*), and deletions (*e.g.*, *pcr1-D1*), although such deletions are designated with deltas in the text for clarity (*e.g.*, *pcr1Δ*). Strains were constructed using standard genetic methods, and were cultured in rich media or minimal media supplemented with specific nutrients and/or G418 at 100 µg per ml (Gutz *et al.* 1974; Forsburg and Rhind 2006).

### Analyses of meiotic recombination

**Figure 1:**
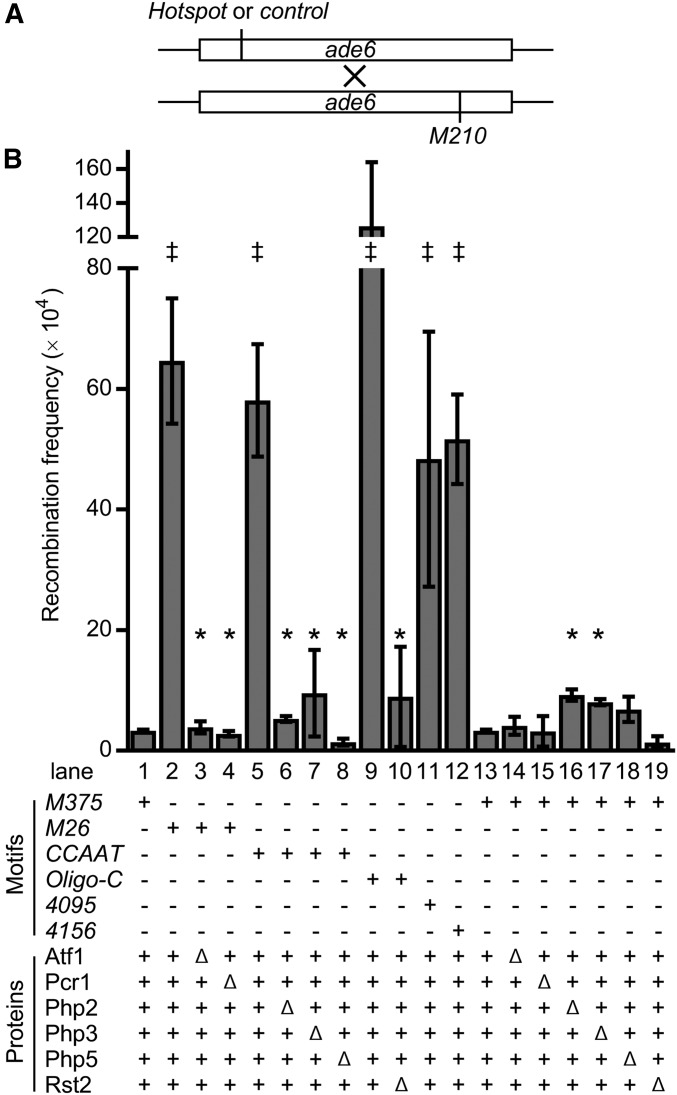
DNA sequence-specific, binding protein-dependent activation of meiotic recombination hotspots. (A) Assay for meiotic recombination. Diagram shows *ade6* ORF (*boxes*) and relative positions of alleles used. Haploid cells harboring a basal recombination control allele (*M375*) or alleles that contain a hotspot DNA sequence motif (*M26*, *CCAAT*, *Oligo-C*, *4095*, and *4156*) were crossed to a strain with a tester allele (*M210*) and haploid meiotic products were scored for frequencies of *ade6^+^* recombinants. (B) Recombinant frequencies from basal control and hotspot crosses in the presence or absence of proteins that bind to the hotspot DNA sequence motifs. Data are mean ± SD from three biological replicates; statistically significant differences (*P* ≤ 0.05% from *t*-test) are shown for hotspot *vs.* control (‡) and hotspot lacking its binding proteins *vs.* with its binding proteins (*). Note that hotspot activation requires both the DNA sequence motif and the protein(s) that bind to that motif, and that ablating these binding proteins does not reduce significantly rates of basal recombination at the control *M375*. The DNA sequences and precise locations of alleles are provided in Table S2; primary data values are provided in Table S4.

Methods to measure rates of intragenic meiotic recombination were as described (Kon *et al.* 1997, 1998), are depicted schematically in Figure 1A, and are summarized here. Heterothallic, haploid strains with different *ade6* alleles (see Table S2 for their DNA sequences) were crossed, and haploid meiotic products (spores) were plated on minimal media that contains or lacks adenine. The titer of Ade^+^ recombinant spore colonies was divided by the titer of all viable spore colonies to yield the recombinant frequency from each cross. Recombinant frequencies (mean ± SD from three independent biological replicates) are plotted in the figures; primary data values from each cross are reported in Table S4 and Table S5.

### Induction of meiosis

The induction of synchronous meiosis by thermal inactivation of Pat1-114^ts^ and the monitoring of meiotic progression were as described (Storey *et al.* 2018), although a different procedure was used to synchronize cells in G_0_ (G_1_) phase of the cell cycle prior to inducing meiosis. The cells were grown to midlog phase (A_595_ = 0.5) at 25° in 167 ml of EMM2 minimal medium containing 1% glucose and 3.75 g/l glutamate as the nitrogen source. Cells were harvested by centrifugation (2500 × *g* for 5 min), washed with ddH_2_O, inoculated into 500 ml of EMM2 media that lacked glutamate, and incubated at 25° for 16 hr to synchronize them in G_0_ phase. Glutamate was added (to 1.0 g/l), cultures were allowed to recover from starvation at 25° for 15 min, and then brought rapidly to 35° (by swirling the flasks in a hot water bath). Cultures were incubated at 35° and samples were collected at the desired time points.

### Mapping of chromatin structure

Our methods for the preparation of mononucleosomes are based on procedures optimized for fission yeast (Lantermann *et al.* 2009, 2010) and that we refined empirically for cells in meiosis. Cell cultures were treated with 0.5% formaldehyde for 20 min to crosslink and stabilize proteins and DNA within chromatin, then the crosslinking reactions were quenched by the addition of glycine to 125 mM. After 10 min, cells were harvested by centrifugation (2500 × *g* for 5 min at 4°), washed with ddH_2_O, collected by centrifugation, and stored as frozen cell pellets at −20° until processed further.

Each preparation of spheroplasts employed formaldehyde-treated cells from 250 ml of culture (∼1.3 × 10^9^ cells). Cells were thawed on ice and resuspended in 6.7 ml of preincubation buffer (20 mM citric acid, 20 mM Na_2_HPO_4_, 40 mM EDTA, pH 8.0) supplemented with 14 µl of β-mercaptoethanol (BME). Cells were collected by centrifugation at 4°, then resuspended in 3.3 ml of spheroplast buffer (1 M Sorbitol, 50 mM Tris-HCl, pH 7.4) supplemented with 2.3 µl of BME and 50 µl of yeast lytic enzyme (100 mg/ml; MP Biomedicals, Santa Anna, CA). Reactions were incubated at 32° for the amount of time determined empirically within each experiment (usually between 30 and 60 min) required to convert essentially all cells to spheroplasts (as judged by light microscopy to monitor changes in cell shape and birefringence, as well as the susceptibility of cells to lysis when exposed to 0.5% SDS). The spheroplasts were collected by centrifugation, were washed with 3.3 ml of the spheroplast buffer without lytic enzyme, and were collected by centrifugation. The spherophasts were then resuspended in 2.5 ml of nuclease reaction buffer (1 M Sorbitol, 50 mM NaCl, 10 mM Tris-HCl pH 7.4, 5 mM MgCl_2_, 1 mM CaCl_2_, 0.75% NP-40) supplemented with 0.5 µl of BME.

For the preparation of mononucleosomes, one-third of each 2.5 ml spheroplast suspension (830 µl) was placed into a tube to serve as an intact-DNA control; two-thirds of each suspension (1660 µl) was placed into another tube for digestion with micrococcal nuclease (MNase). To each sample for digestion we added 3 µl of MNase (0.59 units per microliter; Sigma, Saint Louis, MO), then the no MNase (intact-DNA control) and MNase (nucleosome) samples were incubated in parallel at 37°. After 20 min of incubation, half of the MNase sample (830 µl) was processed, and, at 40 min, the other half of the MNase sample (830 µl) and the intact-DNA control (no MNase) sample (830 µl) were each processed. (Although we titrated the amount and time of MNase digestion for each separate batch of reagents and biological samples to ensure optimal results, we still found that bracketing of reaction times within each experiment was required to ensure the likelihood of obtaining a high proportion of mononucleosomes.) To each tube (830 µl) we added 110 µl of stop buffer (5% SDS, 100 mM EDTA) and 100 µl proteinase K (10 mg/ml; Sigma). The samples were then incubated at 65° for ∼16 hr to reverse crosslinks and digest cellular proteins. Samples of DNA were isolated from intact chromatin and from nucleosome preparations by phenol/chloroform extractions and ethanol precipitation. Both the high MW (undigested) DNA and the mononucleosome-sized (digested) DNA molecules were then isolated by preparative electrophoresis on 2% agarose gels. The DNAs were eluted using Freeze “N” Squeeze DNA gel extraction spin columns (Bio-Rad, Hercules, CA), were recovered by precipitation with isopropanol, were resuspended in 50 µl of TE buffer (10 mM Tris/HCl pH 8.0, 1 mM EDTA), and aliquots were stored at −20° until used as templates for PCR.

**Figure 2:**
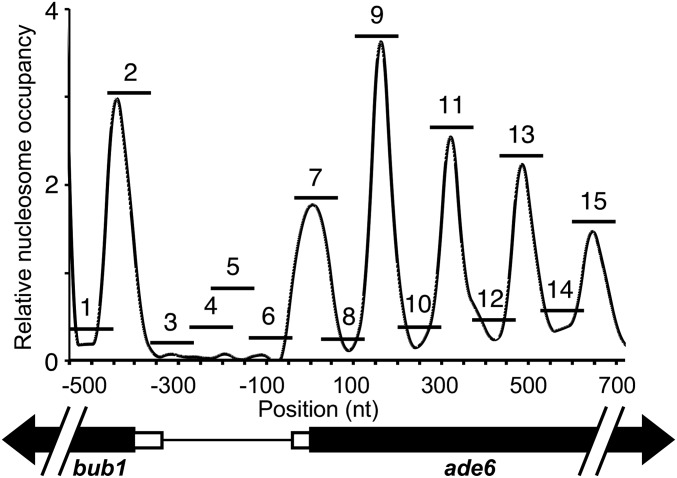
Nucleosome occupancy at *ade6* and design of PCR amplicons used to map chromatin structure. Graph displays our plot of published data on nucleosome occupancy in a *pat1-114* strain background (like that used in our study) prior to entering meiosis (Soriano *et al.* 2013). A diagram of the 1.2 kb region of interest is below, indicating the 5′ UTRs (*open boxes*) and coding regions (*black arrows*) of the *bub1* and *ade6* genes. Relative nucleosome occupancy (GEO study GSE41773, sample GSM1024000, from single-end sequencing of DNA molecules after MNase digestion of chromatin) was plotted with the A of the *ade6* start codon designated as nucleotide (*nt*) position +1. The horizontal bars numbered 1 through 15 indicate the positions of the overlapping PCR amplicons designed for, and used in, this study. See Table S3 for the DNA sequences and coordinates of the PCR primer pairs.

Our approach for the PCR-based mapping of chromatin structure follows those described previously (Sansó *et al.* 2011; Infante *et al.* 2012; García *et al.* 2014; Small *et al.* 2014). Samples of DNA obtained from MNase-digested chromatin were analyzed by real-time, quantitative PCR (qPCR) using All in One qPCR Master Mix (GeneCopeia, Rockville, MD) and the PCR primers listed in Table S3. These primer pairs were based on published global maps of nucleosome occupancy in the fission yeast genome (Lantermann *et al.* 2009, 2010; Soriano *et al.* 2013), and were designed to generate 15 overlapping PCR amplicons that cover a 1.2 kbp region of *ade6* (see Figure 2). The qPCR reactions were carried out using a CFX96 Real Time System (Bio-Rad). Each qPCR reaction (20 µl) contained 0.5 µl of template and 500 nM of forward and reverse primers. Thermocycler parameters were: one cycle at 95° for 10 min; followed by 40 cycles of 95° for 10 sec, 62° for 20 sec, and 72° for 15 sec. In each experiment specificity was confirmed by melting point analyses. For each amplicon, mononucleosomal DNA enrichment was calculated using the ∆∆Ct method (Schmittgen and Livak 2008), with ∆∆Ct = [(Ct_pp_ − Ct_ref_)_No Mnase_ − (Ct _pp_ − Ct_ref_)_+MNase_], where pp refers to the primer pair (1–15), and ref is the *smc5* reference primer pair, in both undigested (No MNase) and digested (+MNase) conditions. Data values for each amplicon under each experimental condition are provided in Table S6. In the figures, each data point (the average from three independent biological replicates) and its SD was plotted at the midpoint of the corresponding amplicon (relative to its position along the *X*-axis). In each case, these positional coordinates were plotted relative to the first nucleotide of the *ade6* start codon (designated as nucleotide position +1). The data points were connected by smoothed curves to represent the inferred positions of nucleosomes, as is commonly done in nucleosome scanning assays that employ a qPCR-based readout [*e.g.*, (Sansó *et al.* 2011; Infante *et al.* 2012; Garcia *et al.* 2014; Small *et al.* 2014]).

### Data availability

The authors state that all data necessary for confirming the conclusions presented in the article are represented fully within the article. Yeast strains and other materials generated by this study are available upon request. All data supporting the conclusions of this study are available within the paper and its supplemental material file. Supplemental material available at FigShare: https://doi.org/10.25386/genetics.9745103.

## Results

Factors that help to position meiotic recombination at hotspots, such as sequence-specific protein–DNA complexes and histone PTMs, each display context-variable penetrance (Wahls and Davidson 2012). Thus to elucidate whether multiple, *cis*-acting, regulatory factors function through a common mechanism, it is necessary to study them within the same chromosomal context. We therefore analyzed the functions of five different hotspot-activating DNA sequence motifs (*M26*, *CCAAT*, *Oligo-C*, *4095*, *4156*; Table 1) (Schuchert *et al.* 1991; Wahls and Smith 1994; Steiner *et al.* 2009, 2011; Foulis *et al.* 2018) located independently at the same site within the *ade6* locus of fission yeast (Figure 1A). In each case, the hotspot alleles were generated by base pair substitutions that create the respective DNA sequence motifs (Table S2), thus maintaining the overall structure and spacing of the locus. The basal recombination control allele (*M375*) and the hotspot alleles were each located near the 5′ end of the *ade6* coding region, mapping within the position of the second nucleosome (+2 nucleosome). Placing the hotspot motifs within a phased nucleosome array allowed us to monitor their impacts on a well-defined, well-organized, chromatin structure.

### Discrete protein–DNA complexes function redundantly to promote meiotic recombination

Haploid strains harboring the basal recombination control (*M375*) or hotspot DNA sequence motifs (*M26*, *CCAAT*, *Oligo-C*, *4095*, *4156*) within *ade6* are each auxotrophic for adenine. These were crossed to a strain with a distal tester allele (*M210*) that is likewise an adenine auxotroph, and the resulting haploid meiotic products were scored for the frequency Ade^+^ (recombinant) spore colonies (Figure 1A). In each case, the hotspot crosses yielded substantially higher recombinant frequencies than the control cross (Figure 1B). For example, the presence of the *M26* DNA sequence motif stimulated recombination ∼20-fold relative to the *M375* control (compare lane 2 to lane 1). This hotspot ratio ranged from 15-fold (for motif *4095*) to 38-fold (for the *Oligo-C* motif), demonstrating that each motif is proficient for promoting recombination (*i.e.*, is active) in this chromosomal context.

Sequence-specific DNA binding proteins have been identified for three of the five motifs (Wahls and Smith 1994; Kon *et al.* 1997; Steiner *et al.* 2009, 2011). Analyses of crosses using null mutants revealed that the stimulation of meiotic recombination by each DNA sequence motif strictly required the proteins that bind to that motif (Figure 1B). Ablating either subunit of the *M26*-binding heterodimer Atf1-Pcr1 abolished hotspot activity of *M26* (compare lanes 3 and 4 to lane 2); removal of the Php2, Php3 or Php5 subunits of the CCAAT box-binding complex abolished hotspot activity of *CCAAT* (compare lanes 6–8 to lane 5); and Rst2 was essential for hotspot activity of *Oligo-C* (compare lane 10 to lane 9). Notably, these null mutant strains were still proficient for meiotic recombination, but not hotspot activity, and yielded recombinant frequencies that were indistinguishable statistically from that of the basal recombination control in wild-type cells (*M375*, lane 1). Furthermore, while ablating these hotspot binding proteins abolished the stimulation of recombination conferred by their respective DNA sequence motifs, the protein deletions did not reduce recombination of the basal recombination control allele, *M375* (compare lanes 14–19 to lane 13). We conclude that the basal recombination machinery (including its catalytic subunit, Rec12) is intact in the respective null mutants. Moreover, because Rec12 (Spo11) and its active site tyrosine are essential for meiotic recombination throughout the fission yeast genome (Sharif *et al.* 2002), including at the *ade6* locus (Kan *et al.* 2011), the hotspot-specific effects indicate that the protein–DNA complexes function specifically to promote the activity of the basal recombination machinery at hotspots. This interpretation is consistent with the fact that the Atf1-Pcr1-*M26* protein–DNA complex promotes the formation of Rec12-catalyzed, recombination-initiating DSBs in its vicinity (Steiner *et al.* 2002).

These findings provided independent confirmation, using a different configuration of test crosses, that multiple different DNA sequence motifs and their binding proteins (transcription factors) each position meiotic recombination at hotspots in fission yeast (Wahls and Smith 1994; Kon *et al.* 1997; Steiner *et al.* 2009, 2011). Moreover, the fact that each *cis*-acting regulatory module is active when placed within the identical chromosomal context allowed us to compare directly whether the different regulatory modules each function through common downstream mechanisms (next three sections).

### Functionally redundant hotspot-activating DNA sequence motifs each induce the remodeling of chromatin structure

It was reported previously, based on Southern blotting, that the *ade6-M26* hotspot allele undergoes chromatin remodeling in meiosis (Yamada *et al.* 2004; Hirota *et al.* 2007). We adopted a well-established MNase and PCR-based approach (Sansó *et al.* 2011; Infante *et al.* 2012; Garcia *et al.* 2014; Small *et al.* 2014) to map chromatin structure of the *ade6* locus at single-nucleosome resolution. Published data on genome-wide nucleosome occupancy (Soriano *et al.* 2013) were used to design 15 pairs of PCR primers that generate tiling, overlapping, amplicons spanning the interval from *bub1* to *ade6* that contains the hotspot DNA sequence motifs (Figure 2, see Table S3 for primer sequences and coordinates). These amplicons were positioned to measure signal intensity within nucleosomes, between nucleosomes, and within a nucleosome-depleted region (NDR).

Cells were treated with formaldehyde to cross-link and stabilize chromatin, then chromatin was digested with MNase, which cleaves DNA preferentially in the linker region between nucleosomes and in NDRs (Lantermann *et al.* 2009, 2010). Following reversal of cross-links, mononucleosomal DNA was purified and used as a template for quantitative, real-time PCR (qPCR). For each *ade6* amplicon, we determined a mononulceosomal DNA enrichment value by comparing signals from untreated (intact) and MNase-treated (mononucleosomal) samples, with internal normalization to an unrelated region of chromosome I (at *smc5*) whose occupancy does not change in meiosis (de Castro *et al.* 2011; Soriano *et al.* 2013). As additional internal controls, we included the shared promoter region between *ade6* and *bub1* and the +1 nucleosome of *bub1*, which is transcribed divergently from *ade6* (Figure 2). Our results with this qPCR-based assay (described below) are consistent with the overall structure of chromatin and the discrete phasing of nucleosomes encompassing the *bub1-ade6* interval, as defined previously using the same MNase sensitivity assay, but with DNA microarray and deep-sequencing readouts of DNA abundance (Lantermann *et al.* 2009, 2010; de Castro *et al.* 2011; Soriano *et al.* 2013).

**Figure 3:**
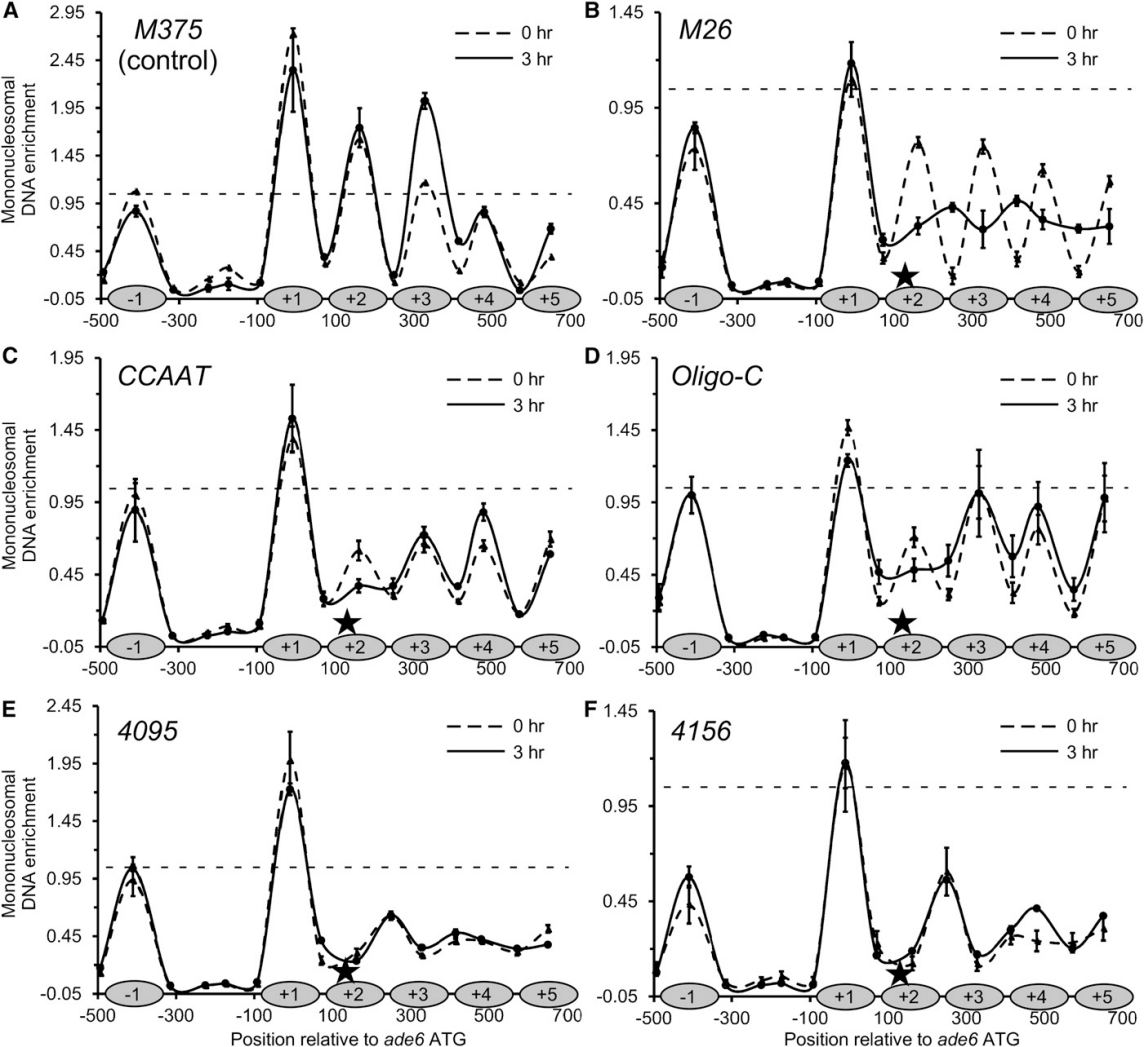
Multiple hotspot-activating DNA sequence motifs each trigger the remodeling of chromatin structure. Plots show chromatin structures at *ade6* before meiosis (*0 hr*) and during meiosis (*3 hr*). Data are mean ± SD from three biological replicates, with internal normalization to a nucleosome in *smc5*. (A) Basal recombination control allele (*M375*). Shaded ovals on the *X*-axis represent protection of DNA by nucleosomes. (B–F) Recombination hotspot alleles (*M26*, *CCAAT*, *Oligo-C*, *4095* and *4156*). The positions of the hotspot-activating DNA sequence motifs (*stars*) and nucleosomes in the basal recombination control [from (A)] are shown on the *X*-axis of each panel for the sake of comparison. Note differences in scale for *Y*-axes (horizontal dashed line at *Y* = 1.0 is included in each panel for visual reference). Tabular data used to generate this figure are provided in Table S5; color-coded plots of superimposed data for different hotspots *vs.* each other and the control are provided in Figure S1.

**Figure 4:**
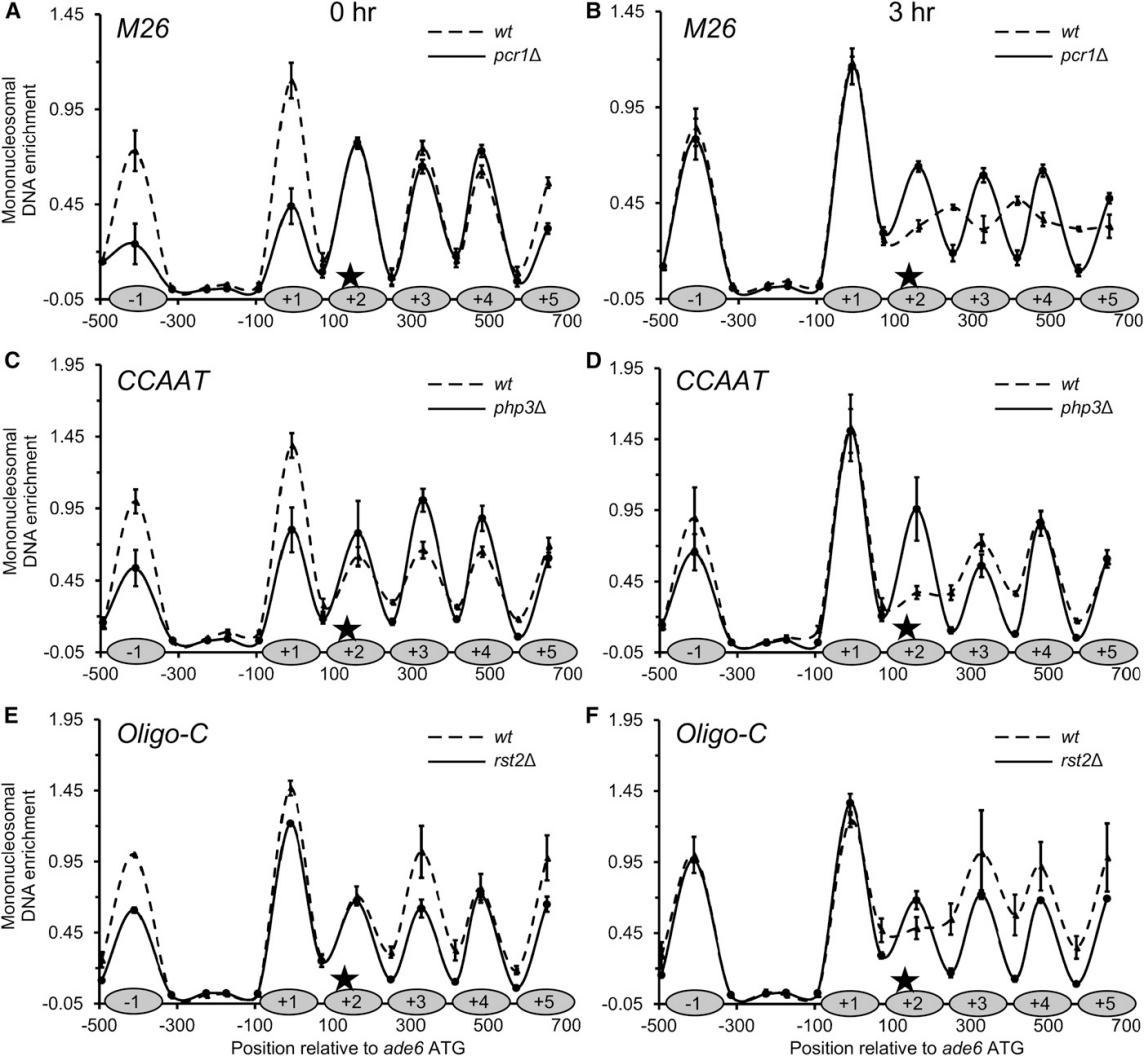
DNA sequence-specific binding proteins regulate chromatin remodeling at sequence-dependent hotspots. Chromatin structures of hotspot alleles before (*0 hr*; A, C, and E) and during meiosis (*3 hr*; B, D, and F) in wild-type cells and in cells lacking the respective DNA binding proteins. Data are mean ± SD from three biological replicates, with internal normalization to a nucleosome in *smc5*. The positions of nucleosomes in the basal recombination control [from (A) of Figure 3] are depicted on each *X*-axis (*shaded ovals*) for the sake of comparison. Note that ablating the hotspot-activating binding proteins restores a more normal phasing of nucleosomes and that this effect is most pronounced at 3 hr of meiosis. Tabular data used to generate this figure are provided in Table S5.

In our figures, we plotted nucleosome positions relative to the start codon of *ade6*. For the sake of reference, the 5′ UTR of the *ade6* transcript is short (46 nucleotides) (Wood *et al.* 2012; Thodberg *et al.* 2019). Except for the differences noted below, all maps conformed to the canonical chromatin structure of fission yeast genes (Lantermann *et al.* 2009, 2010). Nucleosomes were depleted from the shared promoter region between *bub1* and *ade6*; each gene had a well-positioned nucleosome at its transcription start site (denoted in the figures as nucleosome +1 for *ade6* and -1 for *bub1*); and nucleosomes were discretely phased within the *ade6* coding region (Figure 3, Figure 4, and Figure S1).

We sought to define dynamic changes, if any, in chromatin structure during meiosis. We were particularly interested in changes that are present just before, and are thus a potential prerequisite for, recombination-initiating DSBs catalyzed by Rec12 (Spo11) (Cromie *et al.* 2007; Kan *et al.* 2011). To do so, we took advantage of the fact that one can induce highly synchronous meiosis in large cultures of fission yeast by thermal inactivation of the Pat1-114^ts^ repressor of meiosis (Wahls and Smith 1994; Storey *et al.* 2018). We focused our comparative analyses of chromatin structure, in nine different genetic backgrounds, at time points before meiosis (0 hr) and during meiosis (3 hr), with the latter time point being just before maximal induction of DSBs.

#### Chromatin structure of basal control M375:

Except for a difference in the amplitude of signal for nucleosome +3 of *ade6*, the overall structure and phasing of nucleosomes for *ade6-M375* before and during meiosis each superimposed well (Figure 3A). We conclude that meiosis does not trigger any substantial changes in the organization of chromatin at the basal recombination control. The amplitude and positioning for the +1 nucleosome of *bub1* (denoted in the figures as the −1 nucleosome), the low signals within the NDR of the intergenic promoter region between *bub1* and *ade6*, and the amplitude and positioning of nucleosomes within *ade6* each provided controls for the mapping of chromatin structures of *ade6* alleles that harbor hotspot DNA sequence motifs.

#### Chromatin structure of hotspot M26:

For this and other hotspot alleles, the position of the hotspot-activating DNA sequence motif is displayed on the *X*-axis (star), and the inferred positions of nucleosomes as detected in the basal recombination control, *M375*, are provided for comparison (shaded ovals). From cells bearing the *ade6-M26* hotspot, the amplitude of signals for the −1 (*bub1*) nucleosome and for the intergenic NDR region, before and during meiosis, were like those of the control *ade6-M375* (compare Figure 3B to Figure 3A; note difference in scales of *Y*-axes). We infer that the *M26* hotspot motif, which maps within the +2 nucleosome of *ade6*, does not affect substantially chromatin structure outside of the *ade6* transcription unit in which it resides.

Before meiosis, the chromatin structure of the *ade6* coding region harboring the hotspot allele *M26* (Figure 3B, 0 hr) was similar to that of the basal recombination control, *ade6-M375* (Figure 3A). In each case, nucleosomes were well defined and discretely phased within the *ade6* transcription unit. However, there were significant reductions in peak heights for nucleosomes within the *ade6-M26* coding region (Figure 3B) relative to the control (Figure 3A). This can also be seen within each data set by comparing relative peak heights within *ade6* to that of the −1 nucleosome of *bub1*, and by superimposing data sets plotted at the same scale (Figure S1). Meiosis triggered substantial further reorganization of chromatin at *ade6-M26* (Figure 3B, 3 hr). DNA normally protected from MNase digestion by nucleosome +2 (where the *M26* site resides) and downstream nucleosomes became even more accessible to digestion, DNA that is normally in the linker regions became more protected, and there was evidence for the repositioning of nucleosomes (new, minor peaks at +2.5 and +3.5). We conclude that the *M26* hotspot motif regulates meiotically enhanced displacement of nucleosomes, which is consistent with its induction of a more open chromatin structure, as determined previously by Southern blotting (Yamada *et al.* 2004).

Our observation that there were some *M26* hotspot-specific changes in chromatin structure before meiosis (0 hr) was made using cells that had been synchronized in G_0_ (G_1_) phase of the cell cycle, which is necessary to induce synchronous meiosis. To see if such changes were induced by the synchronization process (nitrogen starvation), we analyzed chromatin structures in cells that were growing vegetatively in rich medium (asynchronous, log-phase, mitotic cultures). In these samples, the chromatin structure of the hotspot *ade6-M26* was very similar to that of the basal recombination control, *ade6-M375* (Figure S1). Thus, nitrogen starvation triggers incipient chromatin remodeling at this hotspot before meiosis (0 hr), and there are substantial, meiosis-dependent changes thereafter (3 hr) (Figure 3B).

#### Chromatin structure of hotspot CCAAT:

For the *CCAAT* hotspot motif, the chromatin structures at the −1 (*bub1*) nucleosome and intergenic NDR region were like those of the basal control before and during meiosis (Figure 3C *vs.* Figure 3A). Thus, the *CCAAT* DNA sequence motif, like the *M26* motif, does not seem to affect chromatin structure outside of the *ade6* transcription unit in which it resides.

Before meiosis, the phasing of nucleosomes at *ade6-CCAAT* (Figure 3C, 0 hr) was similar to that of the basal recombination control (Figure 3A). However, there was substantially less protection of DNA within nucleosomes at and flanking the hotspot motif, indicating that the *CCAAT* motif triggers changes in chromatin structure that do not require meiotic factors. These changes surrounding the *CCAAT* motif in *ade6* are attributable to the process of synchronizing cells in G_0_ (G_1_) by nitrogen starvation because the chromatin pattern in vegetative cells was like that of the basal recombination control, *ade6-M375* (Figure S1). Meiosis triggered an additional reduction in the protection of DNA within the +2 nucleosome in which the *CCAAT* motif resides (Figure 3C, 3 hr). We conclude that the *CCAAT* motif, like the *M26* motif, induces chromatin remodeling that encompasses the nucleosome in which it resides and nearby nucleosomes.

#### Chromatin structure of hotspot Oligo-C:

Results for this hotspot motif were similar to those for *M26* and *CCAAT*. Before and during meiosis, there was no evidence of chromatin changes at the −1 (*bub1*) nucleosome and the intergenic NDR (Figure 3D). Before meiosis, there were significant reductions in DNA protection by nucleosomes in the *ade6* coding region, relative to the *M375* control. This property, of changes in chromatin structure preceding meiosis, is shared with the *CCAAT* hotspot (Figure 3C) and the *M26* hotspot (Figure 3B). Meiosis triggered further, *Oligo-C*-dependent remodeling of chromatin structure in its vicinity (Figure 3D), which is also a property shared by each of these three motifs. We conclude that the *Oligo-C* DNA sequence motif, like the other recombination-promoting DNA sequence motifs, regulates changes in local chromatin structure.

#### Chromatin structure of hotspot 4095:

Like the hotspot-activating DNA sequence motifs described above, the *4095* motif had no detectable effect on chromatin structure at the −1 nucleosome and within the NDR, but triggered chromatin remodeling within *ade6* (Figure 3E). The pattern closely resembles that of *M26* in meiosis, with substantial eviction of the +2 and downstream nucleosomes, accompanied by the inferred repositioning of nucleosomes extending to the right of where the *4095* motif resides (new, minor peaks at +2.5 and +3.5). The chromatin maps from before (0 hr) and during meiosis (3 hr) were essentially identical. Analyses of chromatin in vegetative cells revealed the same, *4095*-dependent changes in chromatin structure (Figure S1), suggesting that this hotspot DNA sequence motif induces remodeling constitutively.

#### Chromatin structure of hotspot 4156:

The timing, constellation, and magnitude of chromatin changes elicited by the *4156* motif (Figure 3F), including the remodeling of chromatin in vegetative cells (Figure S1), were like those for *4095*. One difference, relative to all of the other hotspot-activating motifs and the basal recombination control, is that there was significantly reduced protection of DNA within the −1 (*bub1*) nucleosome (Figure 3F), although this was not observed in vegetative cells (Figure S1). This suggests that *4156*-regulated changes in chromatin structure might spread more broadly during meiosis than those triggered by the four other DNA sequence motifs.

In summary, five different meiotic recombination hotspot-activating DNA sequence motifs each regulate the remodeling of chromatin structure locally (Figure 3 and Figure S1). In each case, the chromatin is extensively modified at 3 hr of meiosis, shortly before maximal induction of recombination-initiating DSBs by the catalytic subunit (Rec12/Spo11) of the basal recombination machinery (Cromie *et al.* 2007; Kan *et al.* 2011). For two of the motifs (*4095*, *4156*), the changes are present at both meiotic time points and even in vegetative cells, indicating constitutive disruption of the canonical nucleosome array. For three of the motifs (*M26*, *CCAAT*, *Oligo-C*), remodeling is induced by nitrogen starvation before meiosis (0 hr) and is further enhanced by meiosis (3 hr). For one of these, *M26*, the most substantial changes occur during meiosis. These findings have important implications for molecular mechanisms by which meiotic recombination is positioned throughout the genome (see *Discussion*).

### Hotspot-activating proteins regulate hotspot-specific chromatin remodeling

We next sought to determine whether the sequence-specific, hotspot binding/activating proteins regulate the sequence-dependent chromatin remodeling. We confirmed the ability of null mutant strains to undergo meiosis in a *pat1-114^ts^* genetic background, setting aside the strains in which we could not induce synchronous meiosis (*atf1Δ* and *php5Δ*). The results of chromatin mapping for the three relevant hotspot motifs (*M26*, *CCAAT* and *Oligo-C*), before and during meiosis, and in the presence or absence of their cognate DNA binding proteins, are shown in Figure 4.

#### Effects of Pcr1 protein on chromatin remodeling for hotspot M26:

In a wild-type background, *M26* motif-dependent chromatin remodeling was maximal at 3 hr, with substantial lateral displacement of nucleosomes (Figure 3B and Figure 4B). Under these conditions, the removal of the *M26* binding protein Pcr1 restored the phasing of nucleosome to one like that of the basal recombination control, *ade6-M375* (Figure 4B and Figure 3A, respectively). Pcr1 was required for the increased sensitivity of *M26* hotspot DNA to MNase within canonical nucleosome positions +2 to +5, for the increased protection of DNA between canonical nucleosome positions +1 to +5, and for the apparent repositioning of nucleosomes (new, minor peaks at +2.5 and +3.5).

#### Effects of Php3 protein on chromatin remodeling for hotspot CCAAT:

In a wild-type background, *CCAAT* motif-dependent chromatin remodeling was observed before, and was enhanced further during, meiosis (Figure 3C and Figure 4, C and D). The removal of the *CCAAT* binding protein Php3 restored the chromatin structure of the *CCAAT* hotspot to one similar to that of the basal recombination control. This is most evident during meiosis (Figure 4D, 3 hr). Php3 was also required for the displacement of the +2 nucleosome, and for the increased protection of DNA between the +2 to +5 nucleosomes at the hotspot.

#### Effects of Rst2 protein on chromatin remodeling for hotspot Oligo-C:

In a wild-type background, the *Oligo-C* motif-dependent chromatin remodeling was observed upon nitrogen starvation and was enhanced further by meiosis (Figure 3D and Figure 4, E and F). As was the case for the preceding two hotspot motifs and their binding proteins, removing the *Oligo-C* binding protein Rst2 restored a more normal phasing of nucleosomes, and this was most evident in meiosis (Figure 4F). Rst2 was required for the meiotically enhanced, *Oligo-C*-dependent displacement of the +2 nucleosome, and for the increased protection of DNA between nucleosome positions +1 to +5.

In summary, multiple different DNA sequence-specific, hotspot-activating proteins (transcription factors) each regulate hotspot-specific chromatin remodeling at their DNA binding sites. These changes involve both the nucleosome that contains the hotspot DNA sequence motif and nearby nucleosomes.

### Chromatin remodeling enzyme Snf22 (Snf2/Swi2) is a major effector of sequence-dependent recombination hotspots

**Figure 5:**
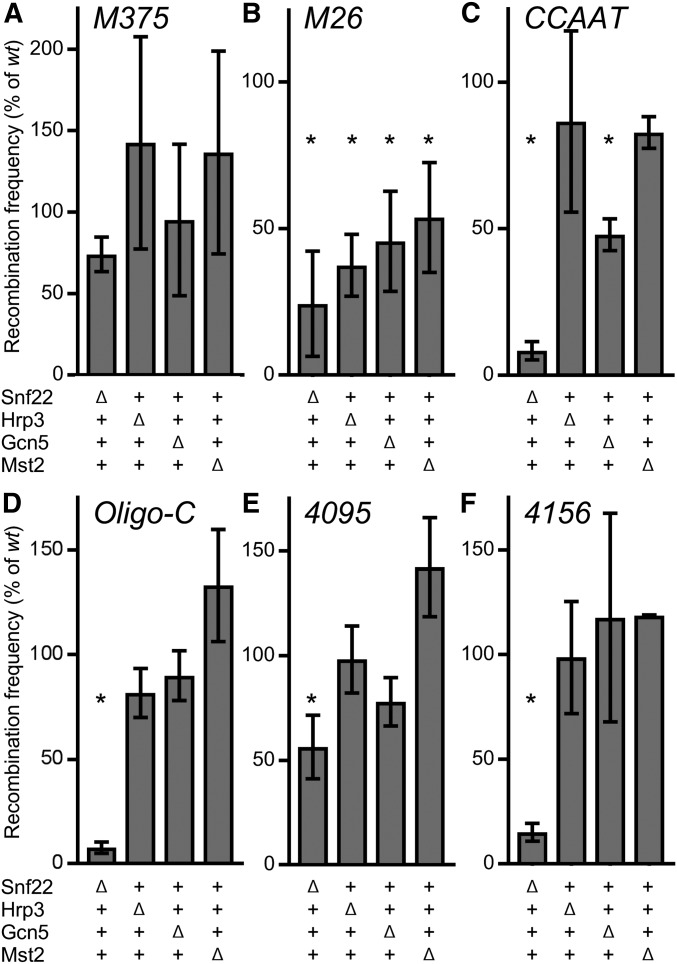
ATP-dependent chromatin remodeling enzyme Snf22 (Snf2/Swi2) is a key regulator of multiple sequence-dependent recombination hotspots. Recombinant frequencies for crosses with basal recombination control [(A) *M375*] and hotspot DNA sequence motifs [(B–F) *M26*, *CCAAT*, *Oligo-C*, *4095*, and *4156*, respectively] were determined as in Figure 1A. Frequencies for the indicated null mutants are expressed as percent relative to frequencies of wild-type cells from crosses conducted in parallel. Data are mean ± SD from three biological replicates; statistically significant differences at *P* ≤ 0.05% (*) from *t*-test are based on recombinant frequencies in both wild type and mutant; note differences in scales of *Y*-axes. Tabular data used to generate this figure are provided in Table S5.

Our experimental system—in which different *cis*-acting regulatory modules are each analyzed within the identical chromosomal context—also allows one to compare directly whether, and the extent to which, various chromatin modifying enzymes regulate diverse classes of DNA sequence-dependent hotspots. There is a plethora of chromatin modifiers, and our choice of which ones to analyze combinatorially (using null mutants and hotspot DNA sequence motifs) was guided by prior findings. For this study, we focused on enzymes already known to be required for efficient chromatin remodeling and for high-frequency recombination at the *ade6-M26* hotspot (Yamada *et al.* 2004, 2013; Hirota *et al.* 2007, 2008). These are the ATP-dependent DNA helicase/chromatin remodeling enzymes Snf22 (of the SWI/SNF family) and Hrp3 (CHD1 family), the SAGA complex histone acetyltransferase catalytic subunit Gcn5, and the Mst2 acetyltransferase of the Mst2/NuA4 complex. We examined the roles of these enzymes in recombination at the five different hotspots (*M26*, *CCAAT*, *Oligo-C*, *4095*, *4156*) and the basal recombination control (*M375*) (Figure 5).

In null mutants lacking Snf22, Hrp3, Gcn5, or Mst2, the recombinant frequencies for the basal recombination control (*M375*) were indistinguishable statistically (at a threshold of *P* ≤ 0.05) from that of wild-type cells (Figure 5A). This indicates that all essential components of the basal recombination machinery are present and functional in the null mutants.

For the *M26* hotspot, recombinant frequencies were significantly lower in *snf22Δ*, *hrp3Δ*, *gcn5Δ*, and *mst2Δ* mutants than in wild-type cells (Figure 5B). Thus, each of the respective chromatin modifying enzymes contributes to high-frequency recombination at *ade6-M26*, with Snf22 being the most important (Snf22 > Hrp3 > Gcn5 > Mst2). Since ablating these proteins did not affect significantly basal recombination (Figure 5A), we conclude that each of these chromatin-modifying enzymes contributes specifically to activation of the *M26* hotspot. These findings are consistent with those reported previously (Yamada *et al.* 2004, 2013; Hirota *et al.* 2007, 2008), providing independent confirmation of results, and serving as a good comparator for additional experiments.

In the *snf22Δ* mutant background, recombination rates were strongly reduced, relative to wild-type cells, for all five different hotspot-activating DNA sequence motifs (Figure 5, B–F). We conclude that diverse hotspot-regulating protein-DNA complexes share an effector mechanism that is mediated by the ATP-dependent chromatin remodeling enzyme Snf22.

Interestingly, while recombination at the *M26* hotspot was attenuated significantly in the *hrp3Δ* and *mst2Δ* mutants, in these mutants there were no significant reductions in recombination for the *CCAAT*, *Oligo-C*, *4095* and *4156* hotspots (Figure 5). Thus, the Hrp3 and Mst2 proteins are required only for activation of one of the five DNA sequence-dependent hotspots, *M26*. Similarly, deletion of *gcn5* did not affect significantly recombination at *Oligo-C*, *4095*, and *4156*, although Gcn5 contributed to high-frequency recombination at *M26* and *CCAAT* (Figure 5).

In summary, the ATP-dependent chromatin remodeling enzyme Snf22 is a crucial mediator of hotspot activation for all five different DNA sequenced-dependent meiotic recombination hotspots analyzed; whereas Gcn5, Hrp3, and Mst2 contribute to the activation of only one or two of the five different classes of hotspots.

## Discussion

### Diverse *cis*-acting regulators employ common mechanism for hotspot activation

The original, evidence-based hypothesis that “Discrete [DNA] sites and their binding proteins could account for the observed regulation of recombination both along the chromosome and during the life cycle” (Wahls and Smith 1994) has been supported by subsequent findings in organisms ranging from fungi to mammals. The hundreds of short, distinct DNA sequence elements shown experimentally to activate meiotic recombination hotspots in fission yeast (Schuchert *et al.* 1991; Wahls and Smith 1994; Kon *et al.* 1997; Steiner *et al.* 2009, 2011; Foulis *et al.* 2018), and those implicated by association in budding yeast [our interpretation of primary data reported by Pan *et al.* (2011)], could account for the positioning of essentially all meiotic recombination hotspots in these two highly diverged species, and, by extension, in others (Steiner *et al.* 2009, 2011; Wahls and Davidson 2010, 2012). This study revealed shared molecular mechanisms by which the functionally redundant, *cis*-acting regulatory elements control recombination locally.

We report that multiple, distinct, recombination hotspot-activating DNA sequence motifs and their binding proteins, where identified (Table 1), each promote recombination by remodeling chromatin structure (Figure 3, Figure 4, Figure 5, and Figure S1). In each case, the hotspot-specific changes in chromatin encompass the nucleosome that contains the regulatory DNA motif, extend to nearby nucleosomes, and precede the time at which the catalytic subunit of the basal recombination machinery (Rec12/Spo11) catalyzes the formation of recombination-initiating DSBs (Sharif *et al.* 2002; Cromie *et al.* 2007; Kan *et al.* 2011). Our finding that chromatin remodeling at five different hotspots precedes recombination is consistent with the observation that meiotically induced chromatin remodeling at the *ade6-M26* hotspot occurs normally in null mutants lacking Rec12 (Spo11) (Hirota *et al.* 2007), even though Rec12 and its active site tyrosine are essential for recombination throughout the fission yeast genome (Sharif *et al.* 2002; DeWall *et al.* 2005; Kan *et al.* 2011). It thus seems clear that neither Rec12 nor Rec12-dependent recombination intermediates contribute appreciably to the hotspot-specific chromatin remodeling that we observed. These findings support a unifying model in which transcription factor-induced changes in chromatin structure help to position the catalytic activity of the basal recombination machinery at hotspots.

### Recombinationally poised epigenetic states

Our mapping of chromatin structure using the highly sensitive, qPCR-based readout revealed increased accessibility of DNA within nucleosomes at the *M26* hotspot before meiosis, relative to the basal recombination control (compare Figure 3B to Figure 3A, see also Figure S1), which had escaped detection by the Southern blotting approach employed previously (Yamada *et al.* 2004, 2013). The finding of some chromatin remodeling before meiosis make sense given that the Atf1-Pcr1 heterodimer binds to *M26* DNA sites in the genome of vegetative cells as well as cells in meiosis (Kon *et al.* 1998; Eshaghi *et al.* 2010; Woolcock *et al.* 2012), promotes the acetylation of histone H3 surrounding the hotspot before meiosis (Yamada *et al.* 2004, 2013), and in vegetative cells affects the chromatin structure of stress-responsive genes whose transcription it regulates (Garcia *et al.* 2014).

We also observed hotspot motif-dependent changes in chromatin structure, relative to the basal recombination control, prior to meiosis for all other DNA sequence-dependent hotspots (Figure 3 and Figure S1). We speculate that changes in such poised epigenetic states might contribute to the modulation of hotspot positioning or strength that are induced by differences in mating type (Parvanov *et al.* 2008), auxotrophies and nutritional states (Abdullah and Borts 2001; Cotton *et al.* 2009), temperature (Fan *et al.* 1995; Zhang *et al.* 2017), and prior freezing (Stahl *et al.* 2016). The fact that transcription factors of fission yeast and budding yeast respond to such intracellular and environmental cues, and that they are rate-limiting for hotspot recombination at their DNA binding sites (there is a protein dose-dependent response) (White *et al.* 1991; Kon *et al.* 1997), supports this idea.

### Global positioning of hotspots by transcription factor-dependent chromatin remodeling

The global distribution of fission yeast DSB hotspots has been mapped (Cromie *et al.* 2007), and analyses of those data revealed that hotspots are directed preferentially to transcription factor binding sites (Wahls and Davidson 2010). Correspondingly, ∼80% of hotspots cluster preferentially at intergenic, promoter-containing regions (Wahls *et al.* 2008; Fowler *et al.* 2014). About 20% of DSB hotspots reside within protein-coding genes (Cromie *et al.* 2007) and occur preferentially in the vicinity of transcription factor binding sites therein (Wahls and Davidson 2010). In every case tested by measuring frequencies of recombination-initiating DSBs or rates of recombination in the presence or absence of candidate regulatory DNA sequences, hotspot activity requires specific DNA sites and their binding proteins [*e.g.*, Figure 1 and (Schuchert *et al.* 1991; Virgin *et al.* 1995; Kon *et al.* 1997; Steiner and Smith 2005; Steiner *et al.* 2009, 2011; Foulis *et al.* 2018)]. Hence, the hotspot-specific, transcription factor-dependent chromatin remodeling mechanisms that we discovered (Figure 3, Figure 4, Figure 5 and Figure S1) are germane to previously reported, genome-wide associations between chromatin accessibility and hotspot positions (de Castro *et al.* 2011; Yamada *et al.* 2013).

We discovered that hotspot-activating DNA sequence motifs can trigger chromatin remodeling before meiosis (as well as during meiosis), and, in some cases, even do so constitutively in vegetative cells (Figure 3 and Figure S1). This striking finding can explain the observation that ∼80% of hotspots colocalize with NDRs that are already present before meiosis and the other 20% are associated with meiotically induced NDRs (de Castro *et al.* 2011). Moreover, our finding that specific chromatin remodeling enzymes are required for the activation of all five different classes of DNA sequence-dependent hotspots (Figure 5) reinforces the conclusion that, in each case, the pathway mechanism proceeds sequentially through transcription factor binding, chromatin remodeling, and stimulation of the basal meiotic recombination machinery.

### Not simply “windows of opportunity”

Our assays of chromatin structure, like those in other studies, involve population-average measurements that infer the distribution of nucleosomes based on the accessibility of DNA to MNase. The interpretation of some changes, like the lateral displacement of nucleosomes (*e.g.*, Figure 3F), is straightforward. Other changes, such as reduced peak height of normally phased nucleosomes (*e.g.*, Figure 3B, 0 hr), might be due to the removal of nucleosomes in a subset of cells, or by looser wrapping of DNA around nucleosomes, or some combination of the two. For example, histone PTMs (such the acetylation of lysine residues) can affect how tightly DNA is wrapped around nucleosomes, and, consequently, sensitivity of that DNA to MNase (Zhao *et al.* 2014; Lorzadeh *et al.* 2016; Jha *et al.* 2017; Kurup *et al.* 2019). Notably, at least 23 different combinations of histone PTMs are enriched preferentially around the *ade6-M26* hotspot at one or more time points of meiosis, relative to basal recombination control (Storey *et al.* 2018). The relevant point here is that DNA sequence-dependent hotspots trigger *many* changes in the underlying constituents of chromatin, and the MNase sensitivity assay reveals only their aggregate impact on the accessibility of DNA within chromatin. For these and the following reasons, we posit that the types of chromatin remodeling induced by the hotspot-activating, sequence-specific binding proteins (not simply “windows of opportunity” associated with NDRs) position recombination at hotspots.

Although most hotspots in the fission yeast genome colocalize with NDRs, the vast majority of NDRs (>90%) do not have an associated hotspot (de Castro *et al.* 2011). Moreover, the NDRs that are associated with hotspots typically have reduced (not fully depleted) nucleosome occupancy (although they are still referred to as NDRs in the literature) (de Castro *et al.* 2011). For example, based on the distribution of a core histone (H3), there is on average only about a 40% reduction of nucleosome occupancy at hotspot centers *vs.* average occupancy genome-wide (Yamada *et al.* 2013). Together, the various association studies have revealed the following, statistically significant trends: the hotspot peaks tend to localize just 5′ of transcription start sites (*i.e.*, in promoter regions) (Wahls *et al.* 2008; Fowler *et al.* 2014); they tend to cluster around transcription factor binding sites (Wahls and Davidson 2010); and they tend to coincide with reduced (but not eliminated) nucleosome occupancy (de Castro *et al.* 2011; Yamada *et al.* 2013).

Each of these associations can also be explained by our data on underlying mechanisms. The hotspot-activating protein-DNA complexes (which in every case defined so far involve transcription factors) each trigger a reduction in the protection of DNA by nucleosomes, without necessarily abolishing the phasing of nucleosomes (Figure 3, B–D). In a subset of cases, there is also evidence for the lateral displacement of nucleosomes, at least in meiosis (Figure 3, B, E, and F). But, in all cases, it seems clear that there is only partial, incomplete hotspot-specific eviction of nucleosomes. In short, the magnitude of hotspot-specific changes that we defined at single-nucleosome resolution, as well as their spatial and temporal patterns at each hotspot analyzed, provide an underlying molecular basis for the genome-wide associations between transcription factor binding sites, changes in chromatin structure and hotspot positions. Together, the findings indicate that transcription factor-specific changes in chromatin—not simply increased accessibility of DNA within NDRs—help to position recombination at hotspots.

### Transcription factors regulate recombination via chromatin modifying enzymes

The remarkably high multiplicity, short lengths, functional redundancy, and context variable penetrance of hotspot-regulating DNA sequences explains why computational analyses have low predictive power for the regulation of recombination by—and can even fail to identify—discrete DNA sequences that are demonstrably recombinogenic (Wahls and Davidson 2012). Similar considerations apply for the multitude of potential downstream effectors, such as chromatin modifying enzymes and histone codes (Storey *et al.* 2018). We reasoned that direct measurements of recombination, for each hotspot *vs.* basal recombination control analyzed under identical conditions, provides the best way to determine whether and the extent to which chromatin remodeling factors regulate diverse classes of recombination hotspots. Positive and negative results of this powerful, well-controlled bioassay are each informative, as exemplified by the following.

The histone acetyltransferases Gcn5 and Mst2, which are required for full activity of *M26*-class hotspots (Yamada *et al.* 2004, 2013; Hirota *et al.* 2007, 2008), had no significant impact on recombination rates at three and four other classes of DNA sequence-dependent hotspots, respectively (Figure 5). This is concordant with the finding that there is only modest association between H3K9ac and essentially no association between H3K14ac with recombination hotspots across the genome, and that mutating the H3K9 acceptor residue has only a very weak impact on the activity of hotspots overall (Yamada *et al.* 2013). Similarly, our finding that the ATP-dependent chromatin remodeling enzyme Hrp3 contributes only to the activation of recombination at *M26*, with no significant impact on recombination for *CCAAT*, *Oligo-C*, *4095* and *4156* (Figure 5), suggests that Hrp3 is a supporting player, rather than a central effector of hotspot activation.

In contrast, the ATP-dependent chromatin remodeling enzyme Snf22 was strongly required for the stimulation of recombination by all five different classes of sequence-dependent hotspots tested (Figure 5). Snf22 is known to be recruited directly to *M26* hotspots, based on chromatin affinity purification of hotspot *vs.* basal recombination control (Storey *et al.* 2018), and is required for both hotspot-specific chromatin remodeling (Yamada *et al.* 2004) and for high-frequency recombination (Figure 5). Together, these findings suggest that transcription factor-regulated remodeling of chromatin structure by Snf22 helps to localize the catalytic activity of the basal recombination machinery at diverse classes of hotspots. Other chromatin remodeling enzymes and histone PTMs that are recruited to hotspots (Storey *et al.* 2018) could be similarly tested with the comparative bioassay. Those that are required for the activation of diverse classes of DNA sequence-dependent hotspots, such as Snf22 (Figure 5), will reveal the subset of chromatin remodelers and histone codes with the greatest impact on the *cis*-acting regulation of recombination, helping to define how they affect the distribution of recombination hotspots genome-wide. Ultimately, as a long-term goal, it would be interesting to test whether the functionally important remodeling enzymes uncovered by the comparative bioassay are recruited to, and regulate via chromatin remodeling, hotspots throughout the genome.

### Conclusions

Our findings support a model in which the binding of transcription factors to their respective DNA sites helps to position most, if not all, meiotic recombination hotspots of fission yeast. The functional redundancy of multiple, sequence-specific protein–DNA complexes converges upon shared chromatin remodeling pathways that help provide the basal recombination machinery (Spo11/Rec12 complex) access to its DNA substrates within chromatin. This mechanism might be conserved in other eukaryotes, including the subset of metazoans that superimpose regulation by another sequence-specific DNA binding protein, Prdm9.
